# The sensitivity of mTORC1 signaling activation renders tissue regenerative capacity

**DOI:** 10.1186/s13619-023-00183-6

**Published:** 2023-12-07

**Authors:** Hanyu Dou, Jianzhou Li, Taomin Huang, Xiaolei Ding

**Affiliations:** 1 Institute of Geriatrics, School of Medicine, Affiliated Nantong Hospital of Shanghai University (The Sixth People’s Hospital of Nantong), Shanghai University, Nantong, 226011 China; 2 Shanghai Engineering Research Center of Organ Repair, Shanghai University, Shanghai, 200444 China; 3 Department of Pharmacy, Eye & ENT Hospital, Fudan University, Shanghai, 200031 China; 4 Department of Dermatology, Huashan Hospital, Fudan University, Shanghai, China

## Abstract

A better understanding of how and why the regenerative capacity differs among species will not only provide insights into the regeneration process but also hold value for the development of regenerative medicine and the improvement of healing procedures. In a recent *Nature* article, Zhulyn et al. identify a critical role played by the activation of mechanistic target of rapamycin complex 1 (mTORC1) signaling in enhancing tissue regenerative capacity in animals.

## Main text

**Fig. 1:**
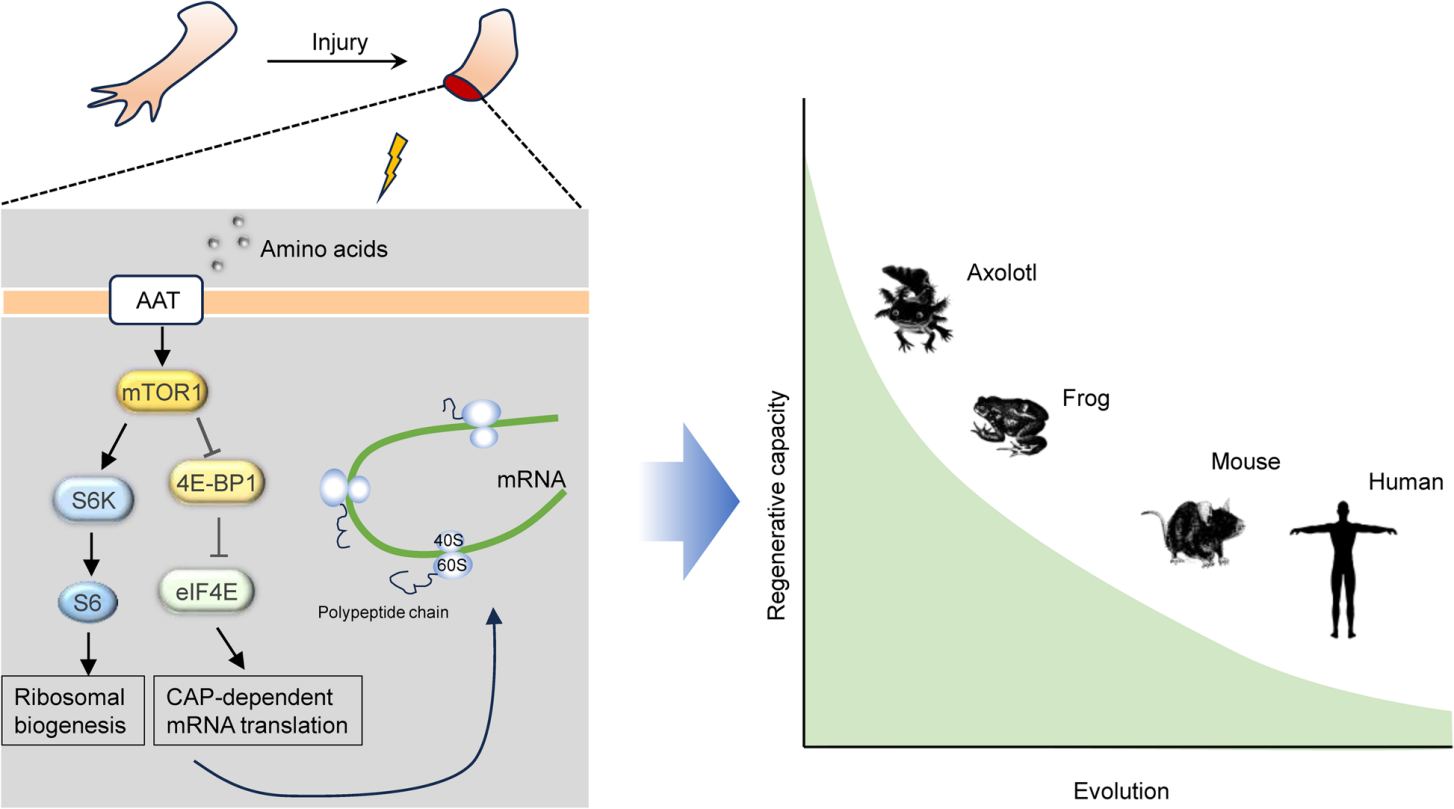
The sensitivity of mTORC1 signaling activation stimulates regeneration in lower vertebrates Upon injury, growth factors and nutrients promote mTORC1 translocation to the lysosome, and mTORC1 activation is rapidly induced in the axolotl amputation model. When activated, mTORC1 controls different processes of protein synthesis, such as cap-dependent translation and ribosome biosynthesis, through phosphorylating 4E-BP1 and S6K1. Zhulyn et al. showed that the rapid protein synthesis mediated by mTORC1 activation drives wound healing and blastema formation, which lead to the consequent complete restoration of the damaged tissues. Thus, manipulation of mTOR signaling activity might facilitate organism regeneration in mammals.

Tissue repair, the process of restoring the integrity and homeostasis of damaged tissues, is fundamental for the survival of all multicellular organisms. Depending on the evolutionary species and life stages, the outcomes of repair can be diverse, ranging from complete restoration of the morphology and function in lower vertebrates (e.g., axolotl and newts) to pathological scarring with a varied degree of fibrosis and unhealing or chronic wounds in mammals (e.g., mouse and human) (Eming et al. 2017) (Fig. 1). Although an enormous effort has been dedicated to investigating regeneration in various model organisms, the mechanisms underlying these differences are complex and not entirely resolved (Bassat and Tanaka 2021). It has been suggested that the regeneration capacities in the lower vertebrates are associated with their rapid healing response and the subsequent formation of the blastema. Blastema contains stem or precursor cells, which can differentiate into multiple cell types and rebuild the damaged tissues. This response needs to enhance the cellular anabolic process, thereby producing the building blocks. However, the precise mechanisms underlying the rapid increase in protein synthesis during tissue repair are not fully understood.

Inspired by the concept, Zhulyn and colleagues set out to explore the molecular mechanisms that regulate the rapid repair response using an axolotl amputation model (Zhulyn et al. 2023). To do so, the authors monitored the changes in translational activity by performing ribosome occupancy analysis. Increases in polysomes were observed in axolotl limb tissues at 24 h post amputation, indicating the initiation of active translation at the early stage of injury. Intriguingly, this induction was absent in the case of mouse digit amputation, correlating to their limited regeneration. Polysome profile analysis by sequencing identified genes encoding proteins about antioxidants and ribosome components that are selectively activated in translation from pre-existing messenger RNAs. These findings imply that the increased protein synthesis may be responsible for the quick healing and later regeneration in axolotls.

The organisms have evolved sophisticated mechanisms to control protein synthesis, given that it is one of the most energy-consuming cellular processes. Mechanistic target of rapamycin complex 1 (mTORC1) signaling is a master controller of cell growth and metabolism by regulating ribosomal biogenesis and protein synthesis (Mossmann et al. 2018). Many of the translational targets identified, which show a rapid increase in axolotl early-stage wound tissues, are known to be directly regulated by mTORC1. To further investigate mTORC1 activation in the injured tissues, the authors analyzed the tissues and observed a rapid induction of mTORC1 signaling activity in the injured tissues of axolotls but not in the mice. Using different inhibitors that block mTORC1 signaling activation to decrease translation, it was found that wound healing and late-stage regeneration are compromised. This suggests that the induction of mTORC1 signaling activity is necessary for regeneration.

What causes the differential mTORC1 responses between axolotl and mouse upon injury? Zhulyn et al. conducted sequence alignment analysis by using sequences of mTORC1 components from various species. The comparison unveiled two insertions within the axolotl mTOR sequence. Structural modeling analysis demonstrated that the insertions could potentially facilitate mTOR to localize to lysosomal membranes, thereby promoting the interactions of the mTOR kinase with other regulatory factors, for instance, Rheb, a key mTORC1-activating protein.

To assess the evolutionary conservation of the effects of the insertions on mTOR signaling activation, the authors engineered mTOR kinase by CRISPR-Cas9 to express the insertions in HEK293T, an immortalized human embryonic kidney cell line. Similar to the observation in the axolotl amputation model, the insertions confer a hypersensitive version of mTORC1 in human cells. This suggests that changes in mTOR structure cause the functional alteration and the insertions may have the potential to enhance translational activity in mammalian cells. However, the functional role of the modification in mammalian tissue repair and morphology merits further investigation.

Overall, the work by Zhulyn et al. underscores the key role of rapid protein synthesis, mediated by mTOR activation, in promoting tissue regenerative capacity. These findings advance our understanding of tissue regeneration and could shed light on the evolutionary loss of the regenerative capacity. The work also raises several questions for future research.

Firstly, it is intriguing to speculate that the variations in regenerative capacities among species may be explained in part by the sensitivity of mTOR signaling activation. The authors identified the specific evolutionary alterations in the molecular structure of mTOR, leading to the increased sensitivity of mTORC1 activation in amphibians. It’s worth noting that mTOR, since its first discovery, has been recognized as an evolutionarily conserved molecule, spanning from yeast to mammalian species. Therefore, a key question arises: what forces drive the structure remolding of mTORC1 during evolution? The combination of multiple experimental model systems will probably pave the way to answering the challenging question.

Secondly, how does mTORC1-mediated translation contribute to blastemal formation? In the past, studies with model organisms have been conventionally conducted to investigate the contribution of transcriptional signals, epigenetic regulations, and chromatin remolding during tissue repair in various tissues and organs (Goldman and Poss 2020). It will be essential to understand the parallelity of proteomic and transcriptional pathways that coordinately control tissue repair by using systemic biology techniques, thereby revealing the underlying mechanisms.

Thirdly, the authors pinpoint the attenuated mTORC1 signaling as a cause of the declined regenerative capacity in the mouse amputation model. It is still unclear how much the structure adaptation of the mTOR kinase described here contributes to the altered regeneration capacity during evolution. In fact, depending on the specific developmental stages, tissues, and organs, humans and the other mammals have considerable regenerative capacity. For instance, at the newborn and fetal stages, most mammals can be completely regenerated (Eming et al. 2017). Particularly, scarless repair occurs in early gestation periods (Fig. 1). Furthermore, some organs in the adults, such as the liver and skin, retain certain regenerative capacities (Espeillac et al. 2011). What specific signaling pathways and regulators that control the regenerative capacity at specific life stages and organs needs to be further identified.

Indeed, we and others have shown that mTOR signaling activation is induced during skin and liver repair (Espeillac et al. 2011; Ring et al. 2023; Wang et al. 2022). Rapid hepatocytes proliferation followed hepatectomy, is associated with mTORC1 activation, as evidenced by enhanced S6 kinase (S6K) phosphorylation. Rapamycin treatment inhibits liver regeneration by blocking S6K activation (Espeillac et al. 2011). Similarly, mTOR signaling is triggered during skin repair and genetically enhanced mTORC1 activity accelerates wound healing progression (Squarize et al. 2010). Furthermore, recent work by Ring et al. reported that the induction of phosphorylation of ribosomal protein S6 (rpS6), a representative marker of mTORC1 activation, can be detected within minutes after skin injury in mice, pigs, and humans (Ring et al. 2023). It is evident that the mechanism underlying the mTORC1 activation during liver and skin repair should be different with the structural adaptation of mTOR kinase described here. These related findings raise the interesting issue of how mTORC1 signaling is activated under different scenarios. It can be through the amino acid sensing pathway as described by Zhulyn et al. or by other cues, for instance, inflammatory stimuli, as described recently in skin repair (Wang and Ding 2022). Further research will have to identify the additional regulators that can enhance the signaling pathway, thereby promoting tissue repair.

## Conclusions

In conclusion, the study by Zhulyn et al. provides a major step forward to our understanding of translational control of tissue regeneration. Selective targeting of mTORC1 signaling for promoting repair may represent an attractive strategy in promoting regenerative processes. However, any consideration of enhancing tissue regeneration by manipulating the process needs to be careful, since hyperactivated mTOR signaling may cause repairing disorders, such as tumor formation (Mossmann et al. 2018). These important findings, along with the advances in ways to control kinase activity spatially and temporally, will offer therapeutic strategies for improving tissue repair efficiency in the clinic.

## Data Availability

Not applicable.
